# Personalised treatment for cognitive impairment in dementia: development and validation of an artificial intelligence model

**DOI:** 10.1186/s12916-022-02250-2

**Published:** 2022-02-01

**Authors:** Qiang Liu, Nemanja Vaci, Ivan Koychev, Andrey Kormilitzin, Zhenpeng Li, Andrea Cipriani, Alejo Nevado-Holgado

**Affiliations:** 1 Department of Psychiatry, University of Oxford, Warneford Hospital, Oxford, OX3 7JX UK; 2 Department of Psychology, University of Sheffield, Sheffield, UK; 3 Institute of Mathematics, University of Oxford, Oxford, UK; 4 Oxford Health NHS Foundation Trust, Warneford Hospital, Oxford, UK; 5 Big Data Institute, University of Oxford, Oxford, UK; 6 Akrivia Health, Oxford, UK

**Keywords:** Dementia, Personalised treatment, Acetylcholinesterase inhibitor, Memantine, Artificial intelligence

## Abstract

**Background:**

Donepezil, galantamine, rivastigmine and memantine are potentially effective interventions for cognitive impairment in dementia, but the use of these drugs has not been personalised to individual patients yet. We examined whether artificial intelligence-based recommendations can identify the best treatment using routinely collected patient-level information.

**Methods:**

Six thousand eight hundred four patients aged 59–102 years with a diagnosis of dementia from two National Health Service (NHS) Foundation Trusts in the UK were used for model training/internal validation and external validation, respectively. A personalised prescription model based on the Recurrent Neural Network machine learning architecture was developed to predict the Mini-Mental State Examination (MMSE) and Montreal Cognitive Assessment (MoCA) scores post-drug initiation. The drug that resulted in the smallest decline in cognitive scores between prescription and the next visit was selected as the treatment of choice. Change of cognitive scores up to 2 years after treatment initiation was compared for model evaluation.

**Results:**

Overall, 1343 patients with MMSE scores were identified for internal validation and 285 [21.22%] took the drug recommended. After 2 years, the reduction of mean [standard deviation] MMSE score in this group was significantly smaller than the remaining 1058 [78.78%] patients (0.60 [0.26] vs 2.80 [0.28]; *P* = 0.02). In the external validation cohort (*N* = 1772), 222 [12.53%] patients took the drug recommended and reported a smaller MMSE reduction compared to the 1550 [87.47%] patients who did not (1.01 [0.49] vs 4.23 [0.60]; *P* = 0.01). A similar performance gap was seen when testing the model on patients prescribed with AChEIs only.

**Conclusions:**

It was possible to identify the most effective drug for the real-world treatment of cognitive impairment in dementia at an individual patient level. Routine care patients whose prescribed medications were the best fit according to the model had better cognitive performance after 2 years.

**Supplementary Information:**

The online version contains supplementary material available at 10.1186/s12916-022-02250-2.

## Background

Dementia is an age-related neurodegenerative syndrome [1], currently affecting approximately 55 million people worldwide, with just under 10 million new cases every year [2]. Medications approved for the treatment of dementia in the US, UK and Canada are the acetylcholinesterase inhibitors (AChEIs; donepezil, rivastigmine and galantamine) and memantine [3]. The use of these medications has been indicated in several guidelines across these countries [4–6]. Generally, AChEIs are recommended for patients with mild to moderate dementia and memantine for patients with moderate to severe dementia [4]. The guidelines are informed by double-blind placebo-controlled randomised trials showing that decline in cognitive performance stabilises for a period of 3–6 months after treatment initiation and have been replicated in a large real-world observational study [7]. However, previous studies that assessed the efficacy of these medications on dementia focused on the average effect of these interventions and did not investigate the potential differences at the level of individual patients [8]. There is a strong movement in clinical research advocating for a more personalised approach in medicine, using more advanced analytical approaches so that nonlinear relationships among multiple factors can be explored together [9].

Recently, artificial intelligence (AI) has been widely applied to dementia research. Deep neural network, one of the most sophisticated machine learning approaches, is commonly used with neuroimaging [10–12] and genetic data [13] as it can make predictions by discovering generalisable nonlinear latent patterns [14, 15] and detect early onset of dementia. Other clinical parameters, such as abnormal alterations in drawing [16], gait [17], and speech [18], have also been informed by AI to effectively monitor brain health and disease progression. In terms of treatment for dementia, AI-driven technologies, such as assistive robots [19] and smart sensors [20], have been developed, but mainly to provide caregiving and management support [21]. Some studies have investigated how to individualise non-pharmacological interventions, e.g. physical exercise recommendation [22], tailored interactive reminiscence session [23], etc., but little to no research has been carried out so far about the personalisation of pharmacological treatment in patients with dementia.

In this study, we aimed to test whether AI-based recommendations based on patient-level information can identify which is the best treatment for each patient and improve their clinical outcome. We tested the specific effect of the 4 recommended drugs for dementia (the three AChEIs and memantine), utilising a large observational dataset of real-world patients. This allowed us to investigate the differential effects of medications by combining demographic data and longitudinal cognitive measures and building a cognitive score prediction model based on deep neural networks to identify the most effective drug for cognitive impairment in dementia at the individual patient level.

## Methods

### Study design and patients

We used de-identified electronic health record (EHR) datasets collected in two UK National Health Service (NHS) Foundation Trusts, namely Oxford Health NHS Foundation Trust (OHFT) and Southern Health NHS Foundation Trust (SHFT). The data are held on the UK CRIS platform [24]. All data used in this study were delivered via the Akrivia Health (https://akriviahealth.com/, Oxford, UK) Data Research Platform under a service agreement between the NHS and Akrivia Health. We selected patients aged 59–102 years with a diagnosis of dementia through both structured International Classification of Disease 10th revision (ICD-10) codes and mentions of dementia diagnosis in the clinical notes by 10 June 2019. The AI model was developed and internally validated on the data from SHFT, while OHFT data were used to externally validate the performance of the model. We identified 3905 individuals from SHFT and another 2899 individuals from OHFT for external validation. The SHFT and OHFT cohorts contributed to a total number of 12,905 and 5296 observations, respectively. In Table 1, we reported the descriptive statistics of cognitive tests in the two cohorts using the Mini-Mental State Examination (MMSE) [25] and the Montreal Cognitive Assessment (MoCA) [26].

**Table 1:** Descriptive statistics of patients from Oxford Health NHS Foundation Trust (OHFT) and Southern Health NHS Foundation Trust (SHFT) based on the time when they received medication for the first time. The data for each Trust is presented separately according to the cognitive scale reported (Mini-Mental State Examination (MMSE) or Montreal Cognitive Assessment (MoCA))

	OHFT (MMSE)	OHFT (MoCA)	SHFT (MMSE)	SHFT (MoCA)
Number of patients	1772	1127	3358	547
Number of observations	3575	1721	12,007	898
Male	37.81%	42.41%	39.70%	42.96%
Age, mean [SD]	81.93 [6.81]	81.72 [6.52]	80.48 [7.61]	80.22 [7.76]
Score, mean [SD]	21.01 [5.11]	17.25 [5.39]	21.10 [5.41]	17.95 [5.51]
Ethnicity (white)	81.66%	55.46%	79.51%	76.05%
Ethnicity (Asian)	0.28%	6.21%	0.33%	0.18%
Ethnicity (black)	0.45%	4.44%	0.21%	0.18%
Ethnicity (others)	0.90%	6.21%	0.27%	NA
Ethnicity (not available)	16.70%	42.86%	19.68%	23.58%
Marital status (married)	40.86%	26.97%	44.16%	40.04%
Marital status (separated)	2.26%	1.77%	4.52%	3.47%
Marital status (single)	4.01%	1.24%	3.21%	3.47%
Marital status (widowed)	28.05%	17.13%	26.18%	21.02%
Marital status (not available)	24.83%	52.88%	21.92%	31.99%

We assert that all procedures contributing to this work comply with the ethical standards of the relevant national and institutional committees on human experimentation and with the Helsinki Declaration of 1975, as revised in 2008. All procedures involving human subjects/patients were approved by the local CRIS oversight committees and are covered by approval for the CRIS database granted by the Oxfordshire and Southern Health Research Ethics Committee. Individual patient consent is not required for this use of anonymised, routine data.

### Sample preparation

Data in UK CRIS consists of both structured fields, such as clinical diagnoses represented by ICD-10 codes, demographic factors and unstructured fields, i.e. clinical notes [27]. We first used a natural language processing (NLP) algorithm [28, 29] to decode the clinical notes into structured data including date of visit, diagnosis, medication and cognitive scale scores such as MMSE and MoCA. Here, we only used NLP to extract cognitive scores stated in the clinical notes as opposed to somehow reconstructing proxy scores. Rule-based NLP was used because we aimed to identify and structure information that often follows certain linguistic patterns in clinical notes. In this situation, defining rules to extract medication prescriptions and cognitive scores was parsimonious in comparison to using statistical learning NER approaches. Moreover, it is more interpretable to clinicians [30]. The macro average F1 score of NLP extractions was 91.21%. Details can be found in Additional file 1: Tables S1 and S2. We then selected the patients who have either a diagnosis of dementia or mentions of dementia in the clinical notes. The selection criteria were further narrowed down to those that had either MMSE or MoCA score recorded. We kept clinical details of all selected patients from their very first observations until the last observations for our longitudinal analysis. The interval for subsequent visits was kept up to 2 years from the initial medication prescription. We excluded patients taking more than one anti-dementia drugs. The patients contributing to only one observation were also excluded since no follow-up data could identify their outcomes. We finally complemented the information of patients with structured demographic factors.

### Predictors and outcomes

Two psychiatrists pre-defined a set of 7 variables from UK CRIS as predictors: age, sex, ethnicity, marital status, duration (time between two continuous observations), cognitive scores (MMSE or MoCA) and prescribed medication, which was considered as a minimal collection for building a personalised prescription model. Age [31] and cognitive score [7] were selected because, among a wide range of clinical and demographic variables, they were found to be independently associated with progression to dementia [32]. Duration can be directly linked to cognitive decline [33], while sex [34], ethnicity [35] and marital status [36] were associated with dementia progression in multiple studies [37–39]. Since we aimed to investigate the specific response to different drugs, prescribed medication was also included as a predictor.

The primary outcome that our model predicted was the cognitive scale score post-drug initiation. MMSE and MoCA were adopted as they are the standard and most often used screening tools for an overall measure of cognitive impairment in both clinical and research settings [40, 41]. Studies have concluded they are sufficiently accurate to detect and monitor cognitive impairment [42, 43]. For both scales, a lower score indicates a more severe cognitive impairment. The treatment that resulted in the smallest decline in cognitive scores between prescription and the next visit was selected as the neural network treatment of choice (NNToC).

### Data analysis

To develop a model capable of identifying best responders given patient’s clinical and demographic information, we built our personalised prescription model using a Recurrent Neural Network (RNN) machine learning architecture (Long Short Term Memory (LSTM) in particular), which has been widely applied in various fields [44–46]. The choice was based on a previous research study [47], where authors showed the LSTM outperformed other traditional models when handling longitudinal EHR data. (We have also conducted analyses using ridge regression, random forest and one-dimensional Convolutional Neural Network, which are detailed in Additional file 1: Fig. S6, Tables S9 and S10.) The core of our model is a two-layer LSTM neural network. We used patients’ cognitive scale change to evaluate the performance of the model at an individual level as this is the outcome reported by the previous studies reporting on the clinical response to these drugs [33, 48, 49].

The front-end stack of fully connected layers can be viewed as a feature extractor across the clinical and demographic information, similar to the principal component analysis, which identifies hidden correlations and patterns and summarises them into a feature vector. The rear-end RNN serves as a cognitive score predictor that captures temporal trends using encoded feature vectors and generates an estimated cognitive score for the four available drugs. Full details of the LSTM model and data pre-processing are reported in Additional file 1: Table S3, Fig. S1 and S2.

We carried out our main analysis using both MMSE and MoCA scores. For the subsequent subgroup analyses, we focused only on MMSE (5130 patients accounting for 15,582 observations from both sites) due to there being considerably fewer instances of MoCA scores (1674 patients with 2619 observations from both sites). The clinical data from SHFT was used for model development and internal validation, while the OHFT data was used for external validation.

#### Analysis using MMSE score

In the first step of the analysis, we identified 3358 patients from SHFT, all of whom had MMSE scores. We randomly selected 2015 [60.01%] individuals for model training and kept the rest for internal validation. For each patient, we randomly selected 4 continuous observations. We did not impute missing values. Instead, missing values were considered as categorical values and incorporated into our model to increase stability and robustness. If a patient had fewer than 4 observations, zero pre-padding (a common process in RNN where zeros are added at the start of the sequence if the length of a sample is shorter than the given standard length) was deployed for null observations. In our case, if a patient had only 1 observation, we padded the first 3 observations with zeros.

In the second step, we predicted the values of cognitive scores for all four available medications. This way, our model produced a contrafactual scenario, whereby we predicted an MMSE score for each medication. We then sorted the predicted scores and selected the medication that generated the highest score to derive the NNToC. Consequently, the patients could be separated into two groups depending on whether they were prescribed NNToC or not. Finally, we evaluated our model and plotted the average change of MMSE given medication prescription across the time of disease. Due to the similar profile of AChEIs, we also conducted analysis where we excluded patients prescribed with memantine and tested our model using AChEIs only.

#### Analysis using MoCA score

Next, we tested our model on patient records with MoCA scores. However, MoCA scores were highly under-reported in SHFT. In this case, we retrained the LSTM on all patients’ data from SHFT and externally validated it on OHFT. On average, each patient contributed to 1.64 observations; thus, we selected 2 continuous observations from each patient for model training.

#### Neural network performance analysis

Additional analyses were carried out to further explore the model robustness and evaluate the recommendation patterns suggested by the neural network. Firstly, we examined the model performance on a random observation rather than focusing on the cognitive score at treatment initiation. Secondly, we tried to address the question of whether the neural network was providing personalised recommendations, or whether it was instead giving better recommendations that generally worked well. To do this, for each patient, we assigned NNToC using the advice for another randomly selected patient. Thirdly, we compared the LSTM model with several other models, namely a more interpretable ridge regression, a traditional random forest machine learning model and a one-dimensional CNN. We pre-processed the data using the same method in our main analysis. For ridge regression and random forest, we flatten the longitudinal features before training [47]. Grid search was used to find the best combination of hyperparameters for every model. Fourthly, due to the imbalance in prescribed medications in the real world, we oversampled the samples in the minority classes by randomly duplicating patients taking minority drugs until each minority class have the same number of patients (patients taking donepezil). We then retrained the model and evaluated the performance. Fifthly, we tried to retrain the model using multitask learning [50], where we fed both MMSE and MoCA scales into one single model. The model then yielded both scores as outputs. Since no patient had both MMSE and MoCA tested on the same visit, i.e. one label was always missing, we included another label indicating which score was missing and only used the non-missing scores to calculate the model loss. Sixthly, instead of using 4 as the sequence length, we retrained the model using only 3 or 2 observations per patient to check the impact of a shorter sequence length. Finally, we adopted permutation feature importance to measure the importance of predictors [51]. Permutation feature importance measures the decrease in a model’s performance when a single predictor is randomly shuffled. Implementations of the model performance analyses are detailed in Additional file 1: Tables S9, S11, S13 and S15.

Data cleaning and data preparation were carried out in R [52]. The neural network model was trained in TensorFlow [53]. The validation and other analyses were carried out using Python [54].

## Results

Our model predicted cognitive scores post-drug initiation for each drug. The treatment that resulted in the smallest decline in cognitive scores between prescription and the next visit was selected as the NNToC. Note that every patient in the study received one of the four possible drugs approved for cognitive symptoms in dementia. Thus, the patients could be separated into two groups depending on whether they were prescribed NNToC or not. We then use patients’ mean cognitive score decline to evaluate the performance of our model.

### Internal validation using MMSE score

**Fig. 1:**
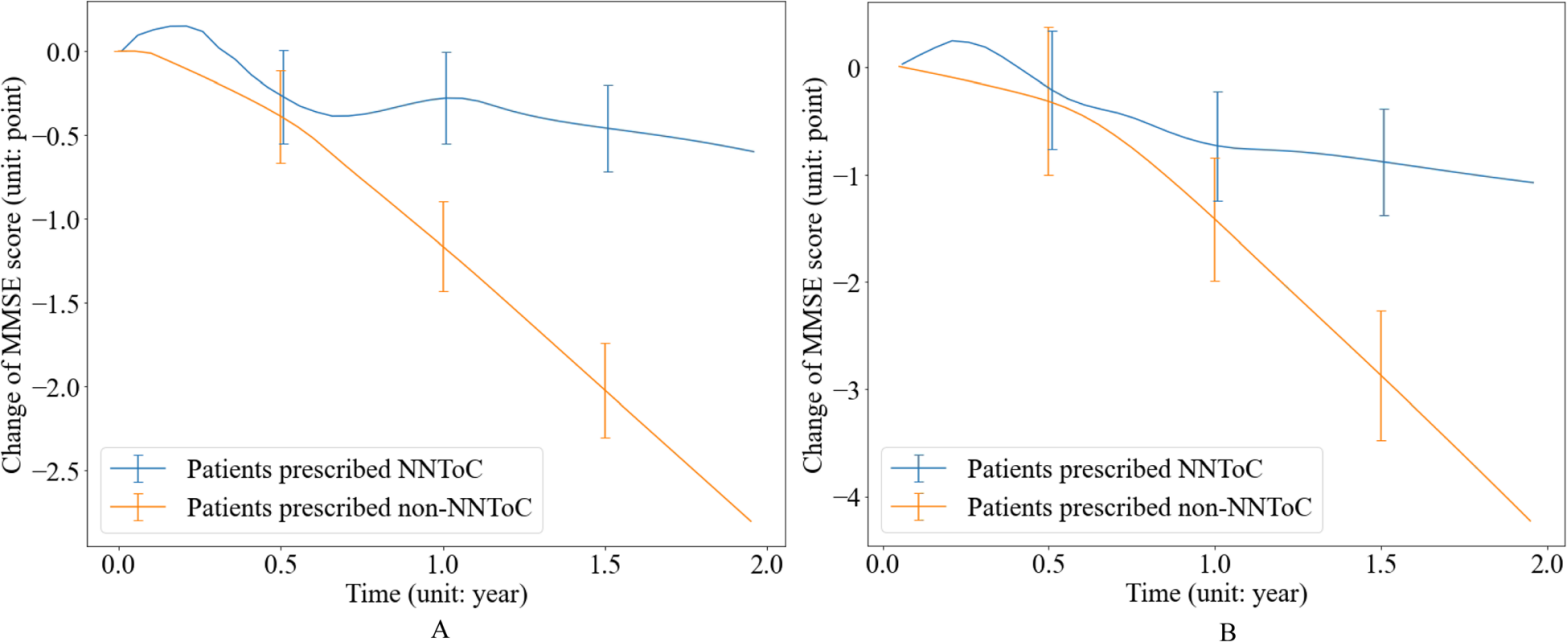
Change of Mini-Mental State Examination (MMSE) score over time. Patients were grouped by whether they were prescribed neural network treatment of choice (NNToC) according to the Long Short Term Memory (LSTM) model. The *X*-axis represents the duration of taking a particular medication, where *x* = 0 means the treatment initiation time. Data shown are mean values, with error bars indicating standard deviation. **A** Internal validation on Southern Health NHS Foundation Trust (SHFT). Two hundred eighty-five [21.22%] patients were prescribed NNToC and reported a smaller MMSE reduction after 2 years compared to the 1058 [78.78%] patients who were not (0.60 [0.26] vs 2.80 [0.28], respectively; *P* = 0.02). **B** External validation on Oxford Health NHS Foundation Trust (OHFT). Two hundred twenty-two [12.53%] patients were prescribed NNToC and reported a smaller MMSE reduction after 2 years compared to the 1550 [87.47%] patients who were not (1.01 [0.49] vs 4.23 [0.60], respectively; *P* = 0.01). A detailed quantitative report of score changes is provided in Additional file 1: Table S5

The rest 1343 [39.99%] patients from SHFT, who were not randomly selected for model training, were used for internal validation. Overall, 285 [21.22%] patients were prescribed NNToC, i.e. they received the medication that generated the highest predicted MMSE scores for their individual case, and 1058 [78.78%] did not get NNToC. We finally plotted the change of MMSE scores in relation to time, as shown in Fig. 1A. The cognitive performance of patients who were not prescribed NNToC declined significantly. In contrast, the patients receiving NNToC declined at a slower rate. Specifically, over the 2 years, their mean [SD] MMSE score declined 0.60 [0.26] points, compared to 2.80 [0.28] who did not receive NNToC (a mean difference of 2.20), which is statistically significant (*P* = 0.02).

### External validation using MMSE score

In terms of external validation, we used the model to evaluate the medication prescription patterns of the OHFT data. We identified 1772 patients with MMSE scores, among which 222 [12.53%] were prescribed NNToC and 1550 [87.47%] were not. The demographics of patients are summarised in Table 2. No significant difference was found between these two cohorts in terms of age, MMSE score and ethnicity; however, there were fewer male (*P* = 0.003) and married patients (*P* = 0.04) who were prescribed NNToC. We also reported the ratio of medications among patients who received NNToC in Additional file 1: Table S4 to show the model was not always prescribing one drug that generally worked well, e.g. memantine.

**Table 2:** Demographics of patients who (i) were prescribed neural network treatment of choice (NNToC) and (ii) were not prescribed NNToC and significance and external validation on Oxford Health NHS Foundation Trust (OHFT) at treatment initiation. *SD* standard deviation, *MMSE* Mini-Mental State Examination, *χ*^2^ chi-square test, *t* Welch’s *t*-test; *P P*-value

	Patients prescribed NNToC (*n* = 222, 12.53%)	Patients prescribed non-NNToC (*n* = 1550, 87.47%)	Significance
Gender (%male)	28.38%	39.10%	*χ*^2^(1) = 9.15, *P* = 0.003
Age, mean [SD]	81.12 [7.01]	82.05 [6.73]	*t*(282.50) = − 1.86 *P* = 0.06
MMSE score, mean [SD]	20.84 [4.89]	21.06 [5.20]	*t*(297.27) = − 0.62 *P* = 0.53
Ethnicity (%Caucasian)	81.08%	81.74%	*χ*^2^(1) = 0.02, *P* = 0.88
Marital status (%married)	34.23%	41.81%	*χ*^2^(1) = 4.30, *P* = 0.04

The change of MMSE score over time is shown in Fig. 1B. We found the trajectories of the cognitive performance of patients from OHFT and SHFT were similar. At the point of initial prescription, all cognitive trajectories had a period of stabilisation lasting approximately 6 months, which indicated treatment effect. After 6 months, the results show that the cognitive performance of patients who were not prescribed NNToC started to decline significantly. The patients not prescribed NNToC generally declined more than the ones taking NNToC in terms of cognitive performance. Specifically, the MMSE score declines of patients taking NNToC and non-NNToC were 0.70 [0.52] and 1.27 [0.59] after 1 year of treatment initiation (*P* = 0.28) and were 1.01 [0.49] and 4.23 [0.60] (a mean difference of 3.22) after 2 years of treatment initiation (*P* = 0.01). A detailed quantified report of MMSE scores on OHFT at selected time points is presented in Additional file 1: Table S5.

For further validation, we excluded patients prescribed with memantine and tested our model on AChEIs only due to the similar profile of these drugs. In this case, the NNToC only include donepezil, rivastigmine and galantamine. We identified 1660 patients who were prescribed AChEIs among which 212 [12.77%] were prescribed NNToC and 1448 [87.23%] were not. The MMSE score declines of patients taking NNToC and non-NNToC were 1.11 [0.47] and 4.29 [0.60] (a mean difference of 3.18) after 2 years of treatment initiation (*P* = 0.02). The MMSE score change and detailed quantified report are presented in Additional file 1: Fig. S3 and Table S6.

### External validation using MoCA score

**Fig. 2:**
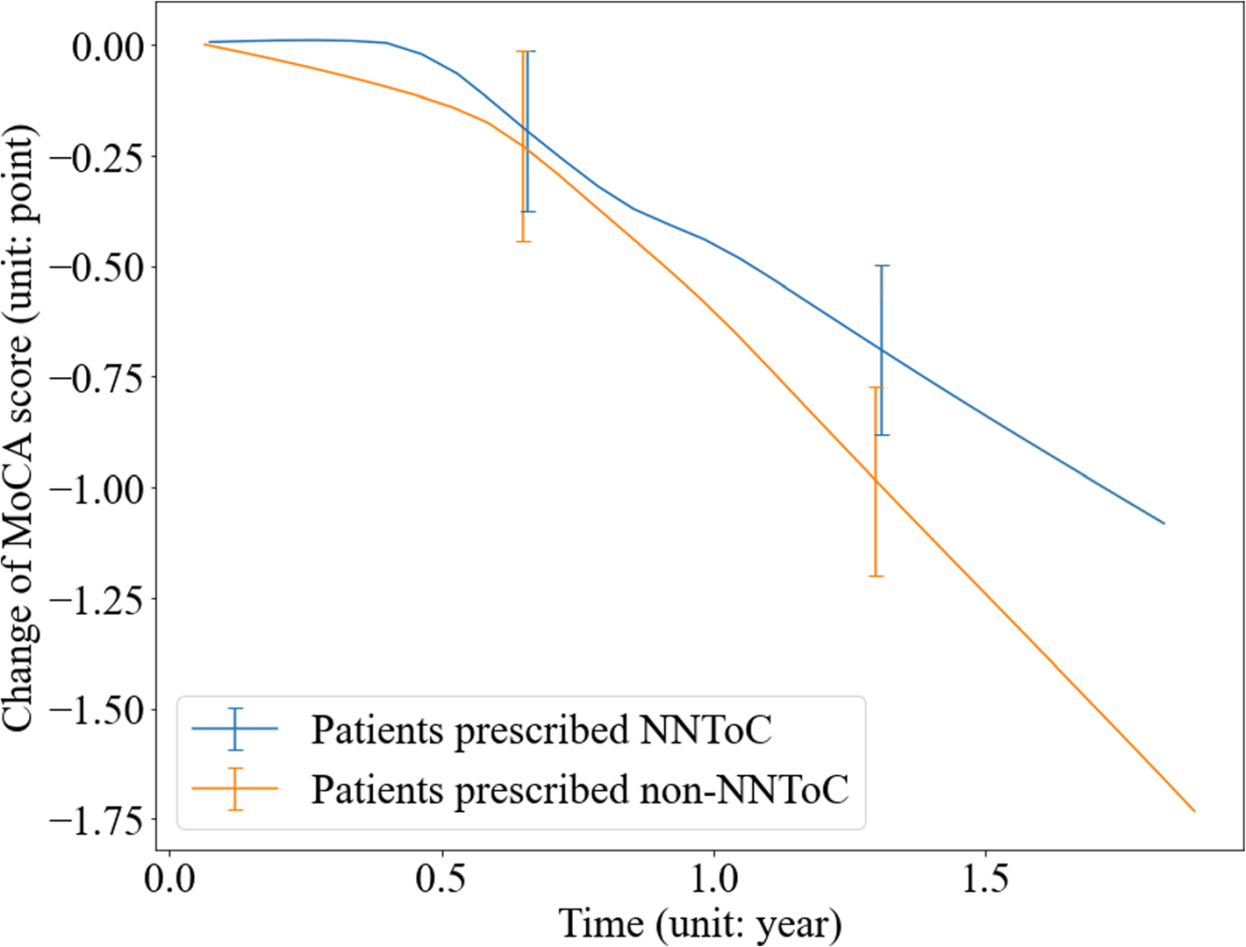
Change of Montreal Cognitive Assessment (MoCA) score over time. Patients were grouped by whether they were prescribed neural network treatment of choice (NNToC) according to the Long Short Term Memory (LSTM) model. The *X*-axis represents the duration of taking a particular medication, where *x* = 0 means the treatment initiation time. Data shown are mean values, with error bars indicating standard deviation. External validation on Oxford Health NHS Foundation Trust (OHFT). One hundred sixty [14.20%] patients were prescribed NNToC and reported a smaller MoCA reduction after 1.75 years compared to the 967 [85.80%] patients who were not (1.03 [0.19] vs 1.56 [0.21], respectively; *P* = 0.09). A detailed quantitative report of score changes is provided in Additional file 1: Table S5

We identified 547 patients with MoCA scores in SHFT for model training and externally validated our model on 1127 patients from OHFT on the observations when a medication was prescribed for the first time, among which 160 [14.20%] were prescribed NNToC and 967 [85.80%] were not. The cognitive performance represented by MoCA scores is shown in Fig. 2. The MoCA score declines of patients who were and were not prescribed NNToC were 1.03 [0.19] and 1.56 [0.21] (a mean difference of 0.53) at 1.75 years (*P* = 0.09).

### Neural network performance analysis

Firstly, the model was tested on a random observation rather than the observation at treatment initiation, as shown in Additional file 1: Fig. S4 and Table S7. After 2 years of treatment initiation, the MMSE score declines of patients who did and did not receive NNToC were 1.92 [0.41] and 4.55 [0.49] (a mean difference of 2.63), respectively (*P* = 0.04). Secondly, we assigned NNToC using the advice for another randomly selected patient. We then recalculated the MMSE scores and the cognitive performance result is reported in Additional file 1: Fig. S5 and Table S8. We observed that the MMSE score change of patients following NNToC dropped significantly 3.26 [0.65] points at 2 years. Thirdly, performance comparison with other models is shown in Additional file 1: Fig. S6 and Table S10. At 2 years, all other models performed similarly, but all worse than the LSTM. However, in the first 6 months, patients who were prescribed NNToC based on the ridge regression had a higher MMSE drop than patients who were not. Fourthly, when the model was trained on the oversampled data, the performance remained almost the same (Additional file 1: Fig. S7 and Table S12). Fifthly, if multitask learning was applied (Additional file 1: Fig. S8 and Table S14), the performance of the model became slightly better when evaluated on MoCA (patients prescribed NNToC had a smaller drop of 0.93 [0.21] points), but worse when evaluated on MMSE (patients prescribed NNToC had a bigger drop of 1.98 [0.70] points). Sixthly, as the sequence length decreased, the difference between the NNToC and non-NNToC groups became less sound at 2 years (Additional file 1: Fig. S9 and Table S16). Particularly, when the model was trained on only 2 observations per patient, the MMSE score of patients who were prescribed NNToC dropped 2.08 [0.65] points, but still smaller than patients who were not at 3.03 [0.61] points (*P* = 0.06).

Finally, we present the permutation feature importance analysis of the LSTM model in Additional file 1: Fig. S10 and Table S17. For the MMSE model, the cognitive score was the absolute dominant predictor (165.52% increase in MAE, 3 times more important than the second important predictor), followed by duration, medication (around 60% increase) and age (36.65% increase). Sex and marital status were much less important (around 5% increase) and ethnicity was not an important predictor (0.10% increase). In terms of the MoCA model, we saw a similar ranking and pattern, but medication was slightly more important than duration.

## Discussion

This paper expands the existing knowledge base on dementia treatment and demonstrates that some individual-to-individual variability exists regarding how patients react to each particular agent used, allowing the identification of individualised treatments that can prolong the positive effects of medication prescription beyond the first 6 months. The model caught the recommendations that were more effective beyond this initial period and the weaker cognitive decline provided by NNToC became more significant later in the disease. Patients prescribed NNToC showed a significantly slower rate of decline and outperformed controls (i.e. patients prescribed a medication different from NNToC) in terms of cognitive performance after 2 years of treatment initiation. In the internal validation on the MMSE cognitive score, the decline was slower by a magnitude of 5 for NNToC relative to the controls. In the external validation sample, we saw a similar, fourfold slower rate of decline in the NNToC group relative to controls. These results demonstrate the value offered by AI-based recommendations in terms of personalised prescription. Currently, the pharmacological treatment with AChEIs is prescribed mainly on the custom of each particular physician [4–6], as it was assumed by default that all AChEIs would have comparable results. In this situation, an informed personalised prescription of AChEIs only has the potential to be more effective. The only approved cognitive drug whose prescription is guided by the particulars of each patient is memantine, given it is most effective in moderate to severe dementia [55], and may better manage behavioural problems [56]. However, our results also show that dementia-based prescription continues having the same overall effect across patients when memantine is taken into account.

Our results about non-personalised prescriptions are consistent with previous studies that after the initial positive effect (typically 3–6 months in duration), patients continue to decline in their cognitive performance [33, 48, 49, 57, 58]. This is important as there is still a debate about whether or not to continue these drugs over a long-term period, considering the marginal cognitive benefit afforded by them when prescribed in a non-personalised manner (0.91 MMSE points at 6 months for Alzheimer’s disease/vascular dementia patients) [59]. These concerns have induced some regulators, e.g. in France, to defund their prescription [60]. We hope that a personalised approach to the prescription of medications in dementia may lead to a proper re-evaluation of the cost-benefit ratio for these drugs.

The neural network was able to generate recommendations even when patients had already been on at least one other anti-dementia drug according to the neural network performance analysis. Notably, the MoCA-based external validation model pointed to a longer stabilisation period of roughly 8 months and a gentler cognitive decline relative to the MMSE-based dataset. This might be because MoCA is capturing earlier cognitive decline through its larger reliance on executive function cognitive domains and it may be less sensitive than MMSE in capturing posterior impairment [61].

In terms of the pattern of recommendations made by the model (ratio of medications), we found that the NNToC highly corresponded to the current guidelines on the prescription of these drugs [4–6]. AChEIs were generally selected as NNToC for patients with mild to moderate severity, whereas memantine was suggested for patients with moderate to severe disease. In the UK, the NICE guidelines do not differentiate AChEIs and only suggest treatment to be started with the drug with the lowest acquisition cost if prescribing an AChEI, which consequently makes donepezil being prescribed more than the other drugs in the real world. However, interestingly, the neural network further avoided suggesting donepezil to men as well as those scoring low on MMSE and tended to instead recommend galantamine, rivastigmine and memantine for these patients. Across disease severity, galantamine, rivastigmine and memantine tended to be NNToC more often. These findings warrant further analysis and validation, with the aim of further refining the current guidelines.

Some potentially important differences between the two clinical cohorts were evident. The patients from the SHFT sample had a lower degree of cognitive decline compared to the OHFT group. One possible explanation is that only 24.38% (432/1772) patients from OHFT were re-examined after a period of 5 months, whereas this proportion for SHFT was 44.58% (1497/3358). According to our previous study [7], patients who were always on one drug benefitted more than patients switching at least once to a different drug. Thus, patients from OHFT may have switched to alternative medications in a timely manner on a follow-up visit, whereas patients from SHFT were able to remain on an agent for a longer time. Another reason might be that patients from OHFT were on average 1.45 years older than those from SHFT. Previous reports have indicated that older age is associated with a faster decline and therefore could account for the larger MMSE score decline in OHFT patients [62, 63].

By comparing the decline of patient cognitive performance in the NNToC and non-NNToC groups, the biggest difference is seen by using the LSTM model trained with 4 observations. Compared to other models, the LSTM explored and captured a higher degree of nonlinearity both in the spatial (predictors) and temporal (observations) space. Although our explanatory analysis using permutation feature shows medication is a strong predictor for the LSTM model (more than 50% increase in MAE), in this case as a general rule for feature importance analysis, it has limited ability to identify the predictor that is most decisive as the predictors cannot be explained individually. It is also worth noting that permutation feature importance only provides a reliable interpretation of the specific model fitted on the training data, but limited insights into the data itself. Therefore, caution is needed when making clinical decisions based on these feature importance results.

A limitation of the study is the defined set of predictors used to build the recommender system. Current predictors include selected demographic variables, cognitive scale scores and medication information that were available through the UK CRIS system. Despite ethnicity being included as a predictor, the distribution was highly skewed towards white-Caucasian (close to 80% in the MMSE samples). Thus, the study may lack the power to detect the potential effect of ethnicity. Although the proposed model showed good performance, the inclusion of a more comprehensive set of predictors (clinical, diagnostic, genetic, omics information) may further improve the capabilities of personalised medication prescription. Neuroimaging data also can contribute volumetric or functional data that may further improve the accuracy of the predictions [64]. Multimodal dataset linkage and big data harmonisation may also offer novel ways to optimise the symptomatic treatment of dementia [65].

Furthermore, it should be noted that widely used assessments, such as MMSE and MoCA, are not necessarily the most accurate. In some studies, MMSE has been shown to be a weaker predictor of dementia severity as compared to other cognitive and functional assessments [66, 67]. Some research suggested that a battery of assessments would be preferable instead of a stand-alone single-administration assessment [68, 69] and this approach may improve the accuracy of our model in the future.

On the other hand, it is known that patients respond differently to drugs depending on the aetiology of the cognitive impairment and the type of dementia, e.g. there is no evidence for efficacy in vascular dementia [70]. In our study, we did not conduct subgroup analyses as 71.74% of the included population were diagnosed with Alzheimer’s disease and the other subtypes of dementia were not large enough to draw meaningful conclusion: only 4.68% had vascular dementia, 4.02% Parkinson’s disease dementia and 0.62% dementia in other diseases (the remaining 18.94% had a diagnosis of unspecified dementia). As a potential future development, we will aim to expand the set of predictors and use individual patient data from randomised control trials combined with observational data to enrich the neural network further. In addition, an online version of the model to be used as a clinical decision support making tool is currently under development.

Finally, in our data, the clinician’s choice of medication was not randomised and may have been influenced by comorbidities and other unknown factors [71]. The conclusion on the effect of a drug in a given person could be compromised by reverse causation, i.e. a particular drug was prescribed more often in subjects that had a priori a worse or better prognosis. We plan to incorporate such factors as predictors to enrich our model in the future. A randomised controlled trial will also be required to test prospectively the utility of the proposed model to establish its real-world efficacy.

Despite the limitations, the findings from external validation have proved the significance of our work and revealed the potential of deep neural networks in the medical science domain. To the best of our knowledge, this is the first personalised prescription model for the pharmacological treatment of cognitive deficits in dementia. This finding suggests the proposed model, and AI-based prescription recommendation in general has the potential to benefit clinical outcomes and can be used by clinical staff as a clinical decision support tool.

## Conclusions

In conclusion, AI-based recommendations produced personalised treatment in dementia, and it was possible to identify at the individual patient level the most effective drug to reduce cognitive impairment over two years. Real-world patients whose prescribed medications were the best fit according to our model had better cognitive performance after 2 years. These results should be replicated in longer-term prospective studies.

## Supplementary Information

**Additional file 1: Figure S1**. An overview of the Long Short Term Memory (LSTM) model. **Figure S2**. Training loss of the Mini Mental State Examination (MMSE) Long Short Term Memory (LSTM) model. **Figure S3**. Change of Mini Mental State Examination (MMSE) score over time when the recommendation drugs were 3 acetylcholinesterase inhibitors (AChEIs; donepezil, rivastigmine and galantamine) only. **Figure S4**. Change of Mini Mental State Examination (MMSE) score over time when a medication was prescribed on a randomly selected visit. **Figure S5**. Change of Mini Mental State Examination (MMSE) score over time when recommendations were randomly shuffled. Figure S6. Change of Mini Mental State Examination (MMSE) score over time when ridge regression, random forest and one-dimensional Convolutional Neural Network (1D CNN) were used for drug recommendation, compared to the Long Short Term Memory (LSTM) model. Figure S7. Change of Mini Mental State Examination (MMSE) score over time when the Long Short Term Memory (LSTM) model was trained on the oversampled data. Figure S8. Change of Mini Mental State Examination (MMSE) and Montreal Cognitive Assessment (MoCA) score over time when recommendations were given by a single Long Short Term Memory (LSTM) model developed using multitask learning. Figure S9. Change of Mini Mental State Examination (MMSE) score over time when the Long Short Term Memory (LSTM) model was trained with fewer observations per patient. Figure S10. Permutation feature importance of the Long Short Term Memory (LSTM) model. Table S1. Validation of the natural language processing (NLP) model performance on the UK Clinical Record Interactive Search (CRIS) data. Table S2. Validation of the natural language processing (NLP) performance on additional categories. Table S3. Implementation details of the Long Short Term Memory (LSTM) model. Table S4. The ratio of prescribed medications. Table S5. Quantified Mini Mental State Examination (MMSE) and Montreal Cognitive Assessment (MoCA) score changes when Long Short Term Memory (LSTM) model was used for drug recommendation. Table S6. Quantified Mini Mental State Examination (MMSE) score changes when the recommendation drugs were 3 acetylcholinesterase inhibitors (AChEIs; donepezil, rivastigmine and galantamine) only. Table S7. Quantified Mini Mental State Examination (MMSE) score changes when a medication was prescribed on a randomly selected visit. Table S8. Quantified Mini Mental State Examination (MMSE) score changes when recommendations were randomly shuffled. Table S9. Implementation details of the ridge regression, random forest and one-dimensional Convolutional Neural Network (1D CNN). Table S10. Quantified Mini Mental State Examination (MMSE) score changes when ridge regression, random forest and one-dimensional Convolutional Neural Network (1D CNN) were used for drug recommendation. Table S11. Implementation details of the Long Short Term Memory (LSTM) model for the oversampled data. Table S12. Quantified Mini Mental State Examination (MMSE) score changes when the Long Short Term Memory (LSTM) model was trained on the oversampled data. Table S13. Implementation details of the Long Short Term Memory (LSTM) model for multitask learning. Table S14. Quantified Mini Mental State Examination (MMSE) and Montreal Cognitive Assessment (MoCA) score changes when recommendations were given by a single Long Short Term Memory (LSTM) model developed using multitask learning. Table S15. Implementation details of the Long Short Term Memory (LSTM) model when the model was trained with fewer observations per patient. Table S16. Quantified Mini Mental State Examination (MMSE) score changes when the Long Short Term Memory (LSTM) model was trained with fewer observations per patient. Table S17. Permutation feature importance scales of the Long Short Term Memory (LSTM) model.

## Data Availability

The data that support the findings of this study (textual documents and structured information) are available by application through the UK CRIS network (https://crisnetwork.co/) via the Akrivia Health (https://akriviahealth.com/, Oxford, UK) Data Research Platform. Owing to the sensitivity of clinical information, access is dependent on receiving research approvals from the NHS trust oversight bodies at Oxford and Southern Health NHS Foundation Trusts.

## Abbreviations

AChEI: Acetylcholinesterase inhibitor
AI: Artificial intelligence
CNN: Convolutional neural network
HER: Electronic health record
ICD-10: International Classification of Disease 10th revision
LSTM: Long Short Term Memory
MMSE: Mini-Mental State Examination
MoCA: Montreal Cognitive Assessment
NHS: National Health Service
NLP: Natural language processing
NNToC: Neural network treatment of choice
OHFT: Oxford Health NHS Foundation Trust
RNN: Recurrent Neural Network
SD: Standard deviation
SHFT: Southern Health NHS Foundation Trust
