# Supplementary material for: Personalised treatment for cognitive impairment in dementia: development and validation of an artificial intelligence model
Source: BMC Med. 2022 Feb 1;20:45. doi: 10.1186/s12916-022-02250-2 (PMC8805393; doi:10.1186/s12916-022-02250-2)
Supplement: Supplementary file 1 — Additional file 1: Figure S1. An overview of the Long Short Term Memory (LSTM) model. Figure S2. Training loss of the Mini Mental State Examination (MMSE) Long Short Term Memory (LSTM) model. Figure S3. Change of Mini Mental State Examination (MMSE) score over time when the recommendation drugs were 3 acetylcholinesterase inhibitors (AChEIs; donepezil, rivastigmine and galantamine) only. Figure S4. Change of Mini Mental State Examination (MMSE) score over time when a medication was prescribed on a randomly selected visit. Figure S5. Change of Mini Mental State Examination (MMSE) score over time when recommendations were randomly shuffled. Figure S6. Change of Mini Mental State Examination (MMSE) score over time when ridge regression, random forest and one-dimensional Convolutional Neural Network (1D CNN) were used for drug recommendation, compared to the Long Short Term Memory (LSTM) model. Figure S7. Change of Mini Mental State Examination (MMSE) score over time when the Long Short Term Memory (LSTM) model was trained on the oversampled data. Figure S8. Change of Mini Mental State Examination (MMSE) and Montreal Cognitive Assessment (MoCA) score over time when recommendations were given by a single Long Short Term Memory (LSTM) model developed using multitask learning. Figure S9. Change of Mini Mental State Examination (MMSE) score over time when the Long Short Term Memory (LSTM) model was trained with fewer observations per patient. Figure S10. Permutation feature importance of the Long Short Term Memory (LSTM) model. Table S1. Validation of the natural language processing (NLP) model performance on the UK Clinical Record Interactive Search (CRIS) data. Table S2. Validation of the natural language processing (NLP) performance on additional categories. Table S3. Implementation details of the Long Short Term Memory (LSTM) model. Table S4. The ratio of prescribed medications. Table S5. Quantified Mini Mental State Examination (MMSE) and Montreal Cognitive Assessment [file 12916_2022_2250_MOESM1_ESM.docx]

Personalised Treatment for Cognitive Impairment in Dementia: Development and Validation of an Artificial Intelligence Model – Supplementary Materials

**Contents**

[Rule Based Natural Language Processing (Table S1, Table S2) 2](#_Toc88090313)

[Annotation Procedure and Model Validation 2](#_Toc88090314)

[Main Analysis using the LSTM Model (Figure S1, Figure S2, Table S3, Table S4, Table S5) 6](#_Toc88090315)

[Drug Recommendation on AChEIs Only (Figure S3, Table S6) 12](#_Toc88090316)

[Receiving Medication on a Random Visit (Figure S4, Table S7) 14](#_Toc88090317)

[Randomly shuffled Recommendations (Figure S5, Table S8) 16](#_Toc88090318)

[Drug Recommendation Using Other Models (Table S9, Figure S6, Table S10) 18](#_Toc88090319)

[Oversampling Patients Prescribed Minority Drugs for Model Training (Table S11, Figure S7, Table S12) 24](#_Toc88090320)

[Training a Single Model to Predict Both Scores Using Multitask Learning (Table S13, Figure S8, Table S14) 27](#_Toc88090321)

[Evaluating the Impact of Training the Model with Fewer Observations per Patient (Table S15, Figure S9, Table S16) 31](#_Toc88090322)

[Permutation Feature Importance (Figure S10, Table S17) 34](#_Toc88090323)

# Rule Based Natural Language Processing (Table S1, Table S2)

The main issue with the unstructured clinical records is that the data of interest, such as psychometric scores, medication and diagnosis information, is locked in the rich textual format. To unlock this information from the raw text format, in the first step of the analysis, we built a natural language processing (NLP) model for named entity extraction. The idea of this model is to statistically process the language used in the clinical notes and identify mentions of medication prescription, cognitive scores and diagnosis information for each patient. In the case of this study, we developed rule-based NLP models in which we define common linguistical constructs how the medication, cognitive score and diagnosis is coded in the clinical texts. The NLP models that we have developed consists of a number of rules that combine concepts of diagnosis outcomes, medication and cognitive scores with lexical and orthography features. We used GATE software (https://gate.ac.uk/) as the analytical framework for text processing, including ManTIME13 for date normalisation.

## Annotation Procedure and Model Validation

In the first step of the analysis, two clinical experts developed a gold corpus that was used to build the natural language processing model. The corpus consisted of 600 annotated Oxford Health notes, where for each note the experts annotated three crucial information: diagnosis, medication, and cognitive scores. The annotation of diagnosis was divided into 5 different subcategories: Alzheimer’s disease, vascular dementia, mixed dementia, Lewy body dementia, and mild cognitive impairment. The medication was equally divided into five different categories: donepezil, rivastigmine, galantamine, acetylcholinesterase inhibitors (broad category), and memantine. Finally, the scale information was subcategorised into two constructs: MMSE and MOCA scores. For each identified concept the time information, that is, the date when the concept occurred, was annotated. Besides annotating the occurrence of concepts in the text, the clinical advisors also indicated who is experiencing this event (e.g. patient, family, other), by whom it is prescribed (secondary care service, primary GP or other), how valid the information is in the text (factual, possible, probable, uncertain, or conditional), and whether it is affirmed or negated. Besides developing a golden corpus of 600 documents, the clinical experts additionally annotated 37 documents that were used to calculate inter-annotator agreement. The NLP model was developed on the set of 360 notes that had 570 mentions of diagnosis, 216 mentions of medication, and 169 mentions of cognitive scores.

The performance of the model was tested on 240 Oxford Health clinical texts that collected 352 mentions of diagnosis, 153 of medication, and 87 of cognitive score information. Additionally, we tested how well the NLP model generalised on Southern Health data on 20 separate notes with 26 mentions of diagnosis, 30 of medication, and 23 of cognitive score information. To test how well the model performs when extracting the information from the text we used a combination of methodologies from “Message understanding conference” (MUC) and “International workshop on semantic evaluation” (SemEval’13). Based on the MUC categories the extractions from the NLP model can be correct (COR), incorrect (INC), partial (PAR - not an identical agreement between gold standard and extraction), missing extraction (MIS – model did not extract the concept), and spurious extraction (SPU – extracted concept that does not exist in the gold standard). Based on this we can calculate the number of possible annotations (POS) in the gold-standard corpus that contribute to the validation score by summarizing correct, incorrect, partial and missing outcomes (true positive + false negative). Equally, we can calculate the total or actual (ACT) number of annotations that our NLP model produced by summarizing correct, incorrect, partial and spurious outcomes (true positive + false positives). Using these two measures we can calculate precision and recall of the system. The precision tells us how many extractions were correct out of the total number of extracted concepts and it is calculated as the ratio between correct (COR) extractions and the actual number of annotations (ACT). The recall indicates the percentage of entities correctly identified in the corpus and is calculated as the ratio between correct (COR) and all possible outcomes (POS). Finally, we can also calculate the overall performance of the model by using harmonic mean between these two values, where $F_{1}=\frac{2*Recall*Precision}{Recall+Precision}$. The NLP model development in this study performed relatively well on all three main categories (see Table S1). In the case of diagnosis, results show that the model identified almost 95% of diagnosis mentions in the texts, while it correctly normalised 85% of them. Much better results are obtained in the case of mentions of medications where our system recognises 98% of medication mentions and correctly normalised 97% of them. Finally, the scale information results in a smaller percentage of recognised values, as approximately 73% of MMSE and MOCA mentions are recognised in the text. Besides calculating the performance of the model, we also estimated how well the two annotators agree when identifying information in the text. This was calculated on the separate 37 clinical texts that were annotated by both clinical experts. However, the final dataset used for development was adjudicated between the two annotators to ensure consistency. Results show almost perfect agreement between them, where not surprisingly they outperform the developed NLP model in the case of diagnosis and scale information. However, the devised NLP model identifies information about the name of the medication slightly better than clinical experts, due to the usage of an extensive dictionary that lists all possible drug names.

Table S1. Validation of the natural language processing (NLP) model performance on the UK Clinical Record Interactive Search (CRIS) data.

| Concept | Precision | Recall | F1-score | Human F1 | F1-Southern |
| --- | --- | --- | --- | --- | --- |
| Diagnosis | 89.6% | 96.3% | 92.8% | 95% | 84.8% |
| Medication | 98.03% | 98.03% | 98.03% | 96% | 78.3% |
| Scale | 92.85% | 74.7% | 82.80% | 100% | 82.6% |

Besides the extraction of the general concepts, we also calculated how well the model performs when extracting the information on the subtype of diagnosis, medication and cognitive scores, agency (who is experiencing problems), relative time information and how valid is the information. Results show that factuality of the information and exact time relation represents a slight problem when annotating and extracting the data, while all other categories reach over 80% of performance (Table S2).

Table S2. Validation of the natural language processing (NLP) performance on additional categories.

|  | NLP model | | | Human annotators | | |
| --- | --- | --- | --- | --- | --- | --- |
| Concept | Diagnosis | Medication | Scale | Diagnosis | Medication | Scale |
| Modality | 71% | 78% |  | 73% | 94% |  |
| Experiencer | 90% | NA |  | 100% | 100% |  |
| Negation | 91% | 95% |  | 90% | 96% |  |
| Opinion by | 93% | 98% |  | 100% | 96% |  |
| Cause | 79% |  |  | 80% |  |  |
| Time relation | 91% | 83% | 62% | 93% | 90% | 95% |

# Main Analysis using the LSTM Model (Figure S1, Figure S2, Table S3, Table S4, Table S5)

An overview of the proposed model is shown in Figure S1.

Figure S1. An overview of the Long Short Term Memory (LSTM) model. Sequences of patient data were first fed into a fully connected layer (FC1) for feature extraction. The extracted features were then fed into LSTM cells and finally another fully connected layer (FC2). Implementation details of the LSTM model are listed in Table S3.

The front-end stack of fully connected layers can be viewed as a feature extractor across the clinical and demographic information of one observation, which identifies hidden correlations and patterns and summarises them into a feature vector. The rear-end recurrent neural network (RNN) serves as a cognitive score predictor that captures temporal trends using feature vectors and generates an estimated cognitive score for all possible drugs. The scores are sorted in descending order and the medication that generated the highest score will be selected as the recommended medication.

The model was trained using supervised learning. The loss function was designed as the deviation between the predicted cognitive score and the ground truth score, i.e., the information observed in the data. In this case, it is the cognitive score recorded on the next visit. The model tried to minimise the loss in an iterative process and adjusted parameters by the back-propagation algorithm. The training loss is shown in Figure S2.


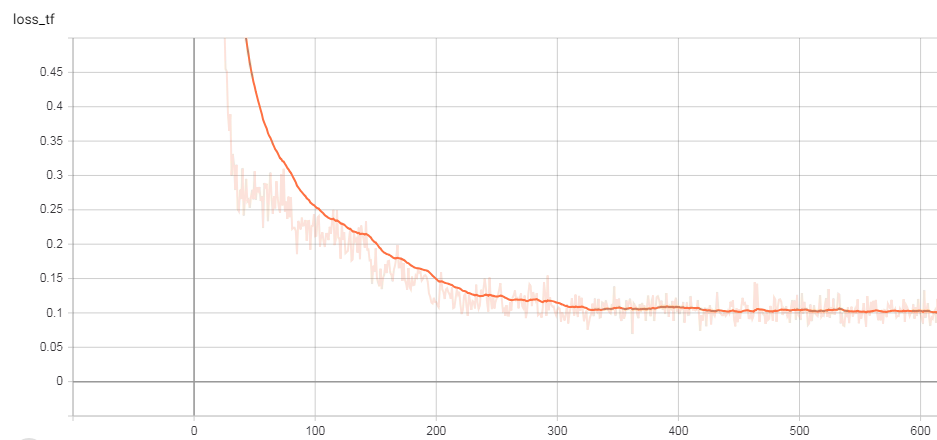


Figure S2. Training loss of the Mini Mental State Examination (MMSE) Long Short Term Memory (LSTM) model. X-axis: training steps. Y-axis: Mean squared error (MSE) loss. Early stopping was applied. Quantity to be monitored was mean absolute error (MAE). The training was stopped 200 epochs after which no improvement was recorded. The model weights were restored from the epoch with the lowest MAE value. Implementation details of the LSTM model are listed in Table S3.

Based on the structured demographic information of the selected cohorts recorded in UK CRIS and the decoded information from clinical notes, age, sex, ethnicity, marital status, current cognitive score, medication and duration were considered as inputs of the model. In terms of data pre-processing, one-hot encoding was deployed for non-numeric covariates (sex, ethnicity, marital status, medication). We did not exclude covariates with missing values. Instead, missing values were considered as categorical values and incorporated into our model to increase stability and robustness. Zero pre-padding was deployed for null observations to make sure the sequence of all patients met the given standard length. All cognitive scores were normalised, i.e., the values were rescaled into a range of [0,1] (divided by the full score), to facilitate model training so that the predicted cognitive score would not be lower than 0 or higher than the full score. Given the aforementioned predictors, the RNN yielded an estimated cognitive score (we rescaled the normalised predicted score back), which indicated what outcome could be achieved by taking a specific medication for a certain duration. The training stopped when the loss (mean squared error of prediction) decline was stable, and the threshold of early stopping was reached.

Table S3. Implementation details of the Long Short Term Memory (LSTM) model.

| Structure | Fully connected layer (FC1) + LSTM layer (LSTM1) + LSTM layer (LSTM2) + fully connected layer (FC2) |
| --- | --- |
| Hyperparameter | FC1 units: 128  FC1 activation: tanh  LSTM1 units: 50  LSTM1 activation: tanh  LSTM2 units: 10  LSTM2 activation: tanh  LSTM2 dropout: 0.4 (MMSE) / 0.5 (MoCA)  FC2 units: 1  FC2 activation: sigmoid  Learning rate: 0.0002  Optimiser: Adam  Loss: mean squared error (MSE)  Early stopping monitor: mean absolute error (MAE)  Early stopping patience: 200 |

Table S4. The ratio of prescribed medications. Patients were grouped according to the Long Short Term Memory (LSTM) model. External validation on Oxford Health NHS Foundation Trust (OHFT) at treatment initiation. N: number of patients. D: donepezil. G: galantamine. R: rivastigmine. M: memantine.

|  | Patients prescribed NNToC (N = 222) | | | | | All patients (N = 1,772) | | | | |
| --- | --- | --- | --- | --- | --- | --- | --- | --- | --- | --- |
|  | N | D | G | R | M | N | D | G | R | M |
| Patients | 222 | 72.07% | 17.12% | 6.31% | 4.50% | 1,772 | 81.55% | 4.80% | 7.34% | 6.32% |
| Male | 63 | 39.68% | 26.98% | 20.63% | 12.70% | 670 | 78.36% | 5.67% | 10.00% | 5.97% |
| Female | 159 | 84.91% | 13.21% | 0.63% | 1.26% | 1,102 | 83.48% | 4.26% | 5.72% | 6.53% |
| Age  60 - 69 | 15 | 80.00% | 6.67% | 6.67% | 6.67% | 88 | 78.41% | 3.41% | 12.50% | 5.68% |
| Age  70 - 79 | 81 | 71.60% | 13.58% | 9.88% | 4.94% | 551 | 79.85% | 4.90% | 8.17% | 7.08% |
| Age  80 - 89 | 108 | 72.22% | 22.22% | 2.78% | 2.78% | 945 | 83.70% | 5.08% | 5.61% | 5.61% |
| Age  >=90 | 18 | 66.67% | 11.11% | 11.11% | 11.11% | 186 | 77.42% | 3.76% | 10.75% | 8.06% |
| MMSE  21-24 | 77 | 75.32% | 15.58% | 6.49% | 2.60% | 591 | 83.93% | 4.57% | 6.94% | 4.57% |
| MMSE  13-20 | 34 | 38.24% | 50.00% | NA | 11.76% | 570 | 81.23% | 6.14% | 7.72% | 4.91% |
| MMSE  <=12 | 6 | NA | 33.33% | NA | 66.67% | 130 | 62.31% | 3.08% | 4.62% | 30.00% |
| White | 180 | 71.11% | 18.33% | 6.67% | 3.89% | 1,447 | 81.69% | 5.18% | 7.39% | 5.74% |
| Married | 76 | 55.26% | 25.00% | 13.16% | 6.58% | 724 | 80.80% | 5.25% | 8.29% | 5.66% |
| Separated, single or widowed | 75 | 74.67% | 21.33% | 2.67% | 1.33% | 608 | 84.54% | 5.10% | 5.26% | 5.10% |

Table S5. Quantified Mini Mental State Examination (MMSE) and Montreal Cognitive Assessment (MoCA) score changes when Long Short Term Memory (LSTM) model was used for drug recommendation. Unit: point. SD: standard deviation. NNToC: neural network treatment of choice. SHFT: Southern Health NHS Foundation Trust. OHFT: Oxford Health NHS Foundation Trust.

|  | Cohort | 0.25 years | 0.5 years | 1 year | 1.5 years | 2 years |
| --- | --- | --- | --- | --- | --- | --- |
| Change of MMSE, treatment initiation, internal validation on SHFT | Patients prescribed NNToC, N=285, mean [SD] | 0.15 [0.11] | -0.21 [0.25] | -0.29 [0.27] | -0.45 [0.26] | -0.60 [0.26] |
|  | Patients prescribed non-NNToC, N=1,085, mean [SD] | -0.10 [0.11] | -0.33 [0.25] | -1.08 [0.27] | -1.94 [0.28] | -2.80 [0.28] |
| Change of MMSE, treatment initiation, external validation on OHFT | Patients prescribed NNToC, N=222, mean [SD] | 0.25 [0.22] | -0.11 [0.50] | -0.70 [0.52] | -0.86 [0.50] | -1.01 [0.49] |
|  | Patients prescribed non-NNToC, N=1,550, mean [SD] | -0.09 [0.28] | -0.27 [0.62] | -1.27 [0.59] | -2.72 [0.60] | -4.23 [0.60] |
| Change of MoCA, treatment initiation, external validation on OHFT | Patients prescribed NNToC, N=160, mean [SD] | 0.01 [0.07] | -0.06 [0.14] | -0.48 [0.19] | -0.89 [0.19] | NA |
|  | Patients prescribed non-NNToC, N=967, mean [SD] | -0.05 [0.09] | -0.14 [0.17] | -0.65 [0.21] | -1.32 [0.21] | NA |

# Drug Recommendation on AChEIs Only (Figure S3, Table S6)


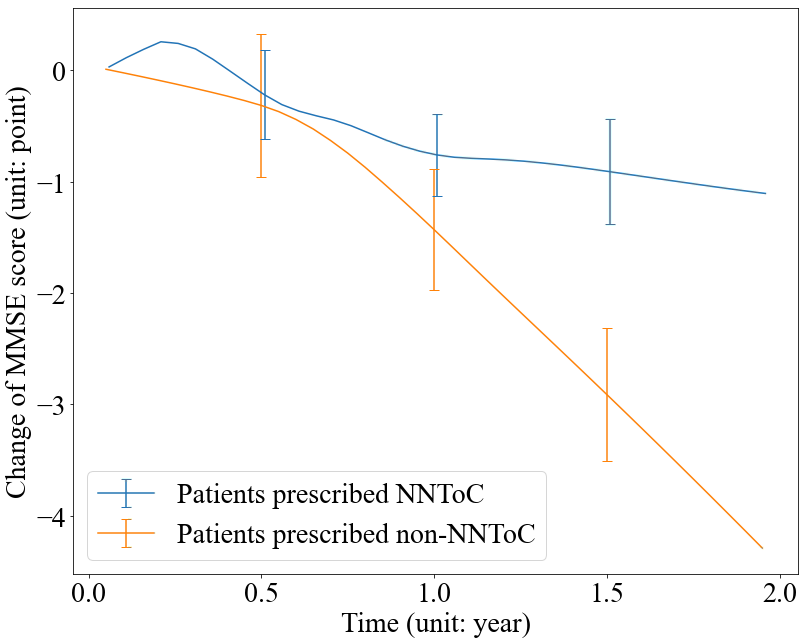


Figure S3. Change of Mini Mental State Examination (MMSE) score over time when the recommendation drugs were 3 acetylcholinesterase inhibitors (AChEIs; donepezil, rivastigmine and galantamine) only. Patients were grouped by whether they were prescribed neural network treatment of choice (NNToC) according to the Long Short Term Memory (LSTM) model. The X-axis represents the duration of taking a particular medication, where $x=0$ means the treatment initiation time. Data shown are mean values, with error bars indicating standard deviation. External validation on Oxford Health NHS Foundation Trust (OHFT). 212 [12.77%] patients were prescribed NNToC and reported a smaller MMSE reduction after two years compared to the 1,448 [87.23%] patients who were not (1.11 [0.47] vs 4.29 [0.60], respectively; P=0.02).

Table S6. Quantified Mini Mental State Examination (MMSE) score changes when the recommendation drugs were 3 acetylcholinesterase inhibitors (AChEIs; donepezil, rivastigmine and galantamine) only. External validation on Oxford Health NHS Foundation Trust (OHFT) at treatment initiation. Unit: point. SD: standard deviation. NNToC: neural network treatment of choice.

| Cohort\Year | 0.25 | 0.5 | 1 | 1.5 | 2 |
| --- | --- | --- | --- | --- | --- |
| Patients prescribed NNToC, N=212, mean [SD] | 0.26 [0.16] | -0.11 [0.36] | -0.73 [0.37] | -0.89 [0.46] | -1.11 [0.47] |
| Patients prescribed non-NNToC, N=1,448, mean [SD] | -0.09 [0.26] | -0.27 [0.58] | -1.29 [0.55] | -2.76 [0.59] | -4.29 [0.60] |

# Receiving Medication on a Random Visit (Figure S4, Table S7)


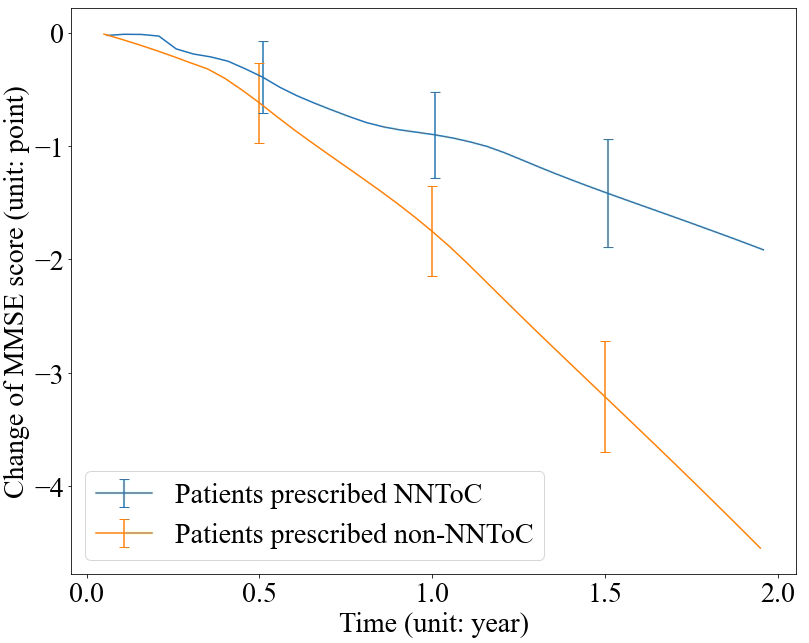


Figure S4. Change of Mini Mental State Examination (MMSE) score over time when a medication was prescribed on a randomly selected visit. Patients were grouped by whether they were prescribed neural network treatment of choice (NNToC) according to the Long Short Term Memory (LSTM) model. The X-axis represents the duration of taking a particular medication, where $x=0$ means the time from when a medication was prescribed on a randomly selected visit. Data shown are mean values, with error bars indicating standard deviation. External validation on Oxford Health NHS Foundation Trust (OHFT). 410 [23.14%] patients were prescribed NNToC and reported a smaller MMSE reduction after two years compared to the 1,362 [76.86%] patients who were not (1.92 [0.41] vs 4.55 [0.49], respectively; P=0.04).

Table S7. Quantified Mini Mental State Examination (MMSE) score changes when a medication was prescribed on a randomly selected visit. External validation on Oxford Health NHS Foundation Trust (OHFT). Unit: point. SD: standard deviation. NNToC: neural network treatment of choice.

| Cohort\Year | 0.25 | 0.5 | 1 | 1.5 | 2 |
| --- | --- | --- | --- | --- | --- |
| Patients prescribed NNToC, N=410, mean [SD] | -0.03 [0.12] | -0.32 [0.29] | -0.88 [0.37] | -1.36 [0.47] | -1.92 [0.41] |
| Patients prescribed non-NNToC, N=1,362, mean [SD] | -0.15 [0.14] | -0.51 [0.32] | -1.63 [0.39] | -3.07 [0.48] | -4.55 [0.49] |

# Randomly shuffled Recommendations (Figure S5, Table S8)


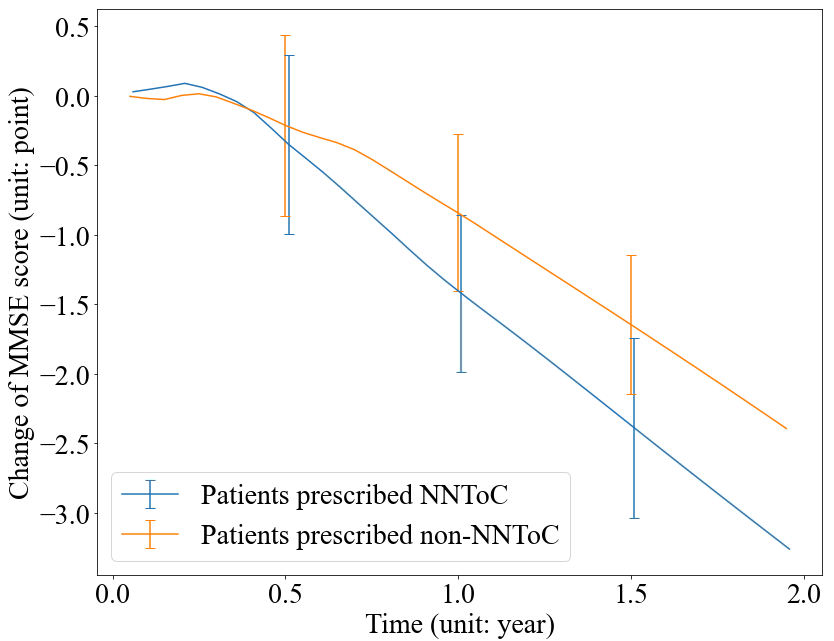


Figure S5. Change of Mini Mental State Examination (MMSE) score over time when recommendations were randomly shuffled. Patients were grouped by whether they were prescribed neural network treatment of choice (NNToC) according to the Long Short Term Memory (LSTM) model. The X-axis represents the duration of taking a particular medication, where $x=0$ means the treatment initiation time. Data shown are mean values, with error bars indicating standard deviation. External validation on Oxford Health NHS Foundation Trust (OHFT). The MMSE of 212 [12.77%] patients who were prescribed NNToC dropped significantly 3.26 [0.65] points.

Table S8. Quantified Mini Mental State Examination (MMSE) score changes when recommendations were randomly shuffled. External validation on Oxford Health NHS Foundation Trust (OHFT). Unit: point. SD: standard deviation. NNToC: neural network treatment of choice.

| Cohort\Year | 0.25 | 0.5 | 1 | 1.5 | 2 |
| --- | --- | --- | --- | --- | --- |
| Patients prescribed NNToC, N=222, mean [SD] | 0.09 [0.26] | -0.23 [0.58] | -1.32 [0.57] | -2.29 [0.64] | -3.26 [0.65] |
| Patients prescribed non-NNToC, N=1,550, mean [SD] | 0.01 [0.26] | -0.16 [0.58] | -0.77 [0.57] | -1.56 [0.51] | -2.39 [0.50] |

# Drug Recommendation Using Other Models (Table S9, Figure S6, Table S10)

Table S9. Implementation details of the ridge regression, random forest and one-dimensional Convolutional Neural Network (1D CNN).

| Model | Hyperparameter |
| --- | --- |
| Ridge regression | L2 regularisation  Regularization strength α: 1.0 |
| Random forest | n_estimators: 10  max_depth: 3  min_samples_split: 2  min_samples_leaf: 10  bootstrap: True  criterion: squared_error |
| One-dimensional Convolutional Neural Network (1D CNN) | Structure: fully connected layer (FC1) + Conv layer + fully connected layer (FC2) + fully connected layer (FC3)  FC1 units: 128  FC1 activation: tanh  Conv filters: 64  Conv kernel_size: 3  Conv padding: valid  Conv strides: 1  Conv activation: tanh  FC2 units: 32  FC2 activation: tanh  FC2 dropout: 0.4  FC3 units: 1  FC3 activation: sigmoid  Learning rate: 0.0002  Optimiser: Adam  Loss: mean squared error (MSE)  Early stopping monitor: mean absolute error (MAE)  Early stopping patience: 200 |


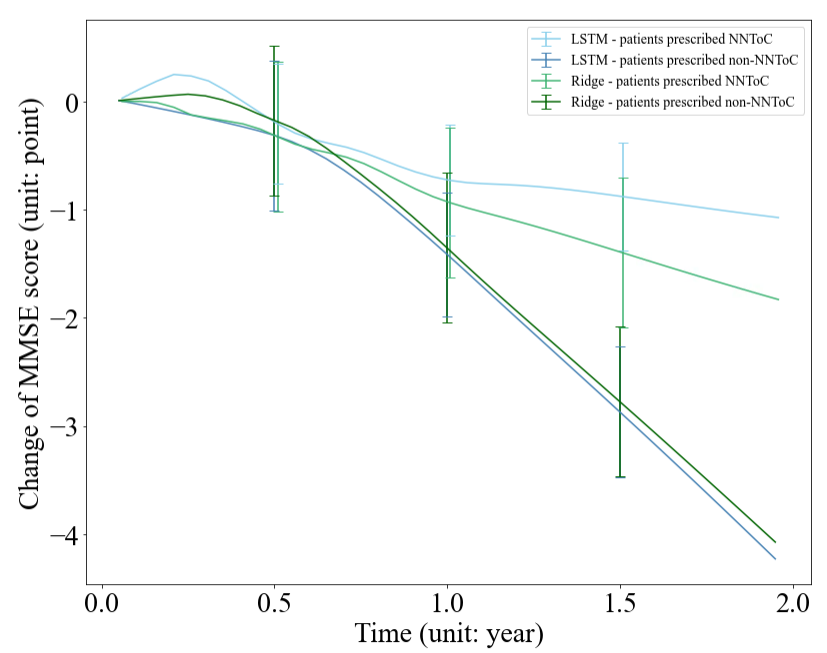

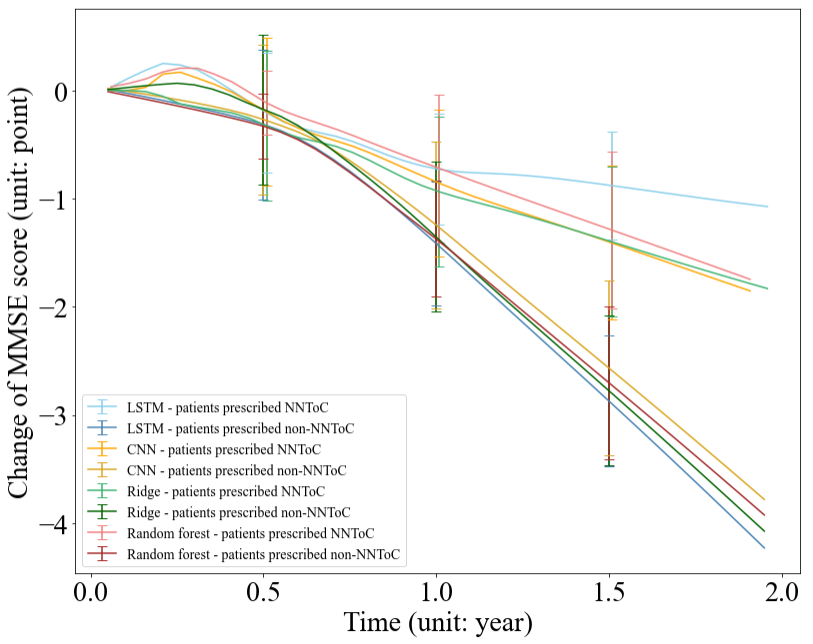

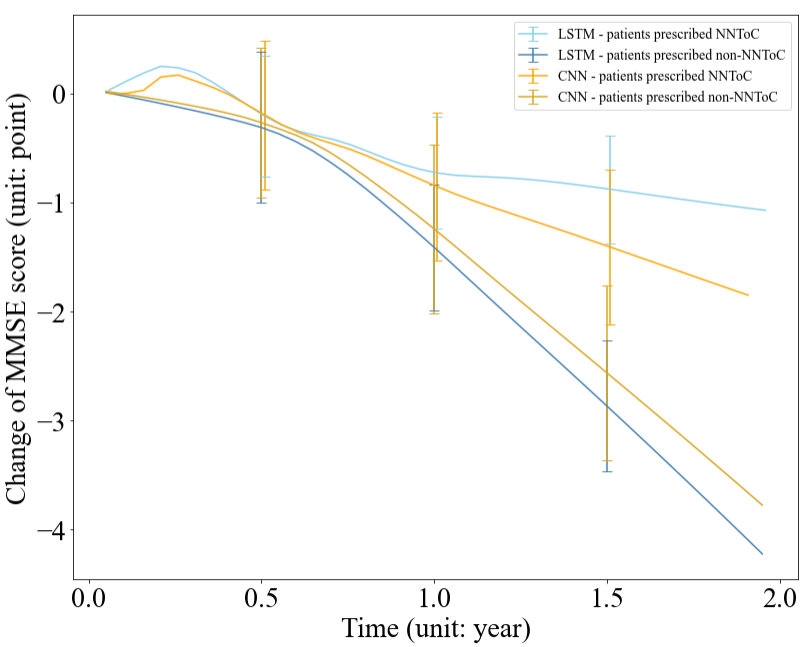

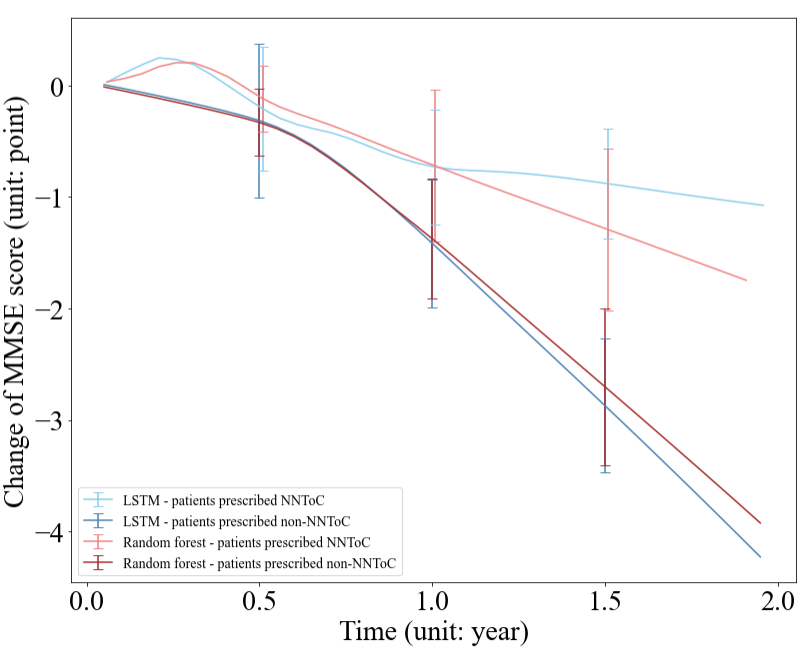


C

B

D

A

Figure S6. Change of Mini Mental State Examination (MMSE) score over time when ridge regression, random forest and one-dimensional Convolutional Neural Network (1D CNN) were used for drug recommendation, compared to the Long Short Term Memory (LSTM) model. External validation on Oxford Health NHS Foundation Trust (OHFT). The X-axis represents the duration of taking a particular medication, where $x=0$ means the treatment initiation time. Data shown are mean values, with error bars indicating standard deviation. (A) Patients were grouped by whether they were prescribed neural network treatment of choice (NNToC) according to the ridge regression model. 101 [5.70%] patients were prescribed NNToC and reported a smaller MMSE reduction after two years compared to the 1,671 [94.30%] patients who were not (1.83 [0.69] vs 4.07 [0.69], respectively; P=0.04). (B) Patients were grouped by whether they were prescribed neural network treatment of choice (NNToC) according to the random forest model. 708 [39.95%] patients were prescribed NNToC and reported a smaller MMSE reduction after two years compared to the 1,064 [60.05%] patients who were not (1.76 [0.49] vs 3.92 [0.70], respectively; P=0.04). (C) Patients were grouped by whether they were prescribed neural network treatment of choice (NNToC) according to the 1D CNN model. 482 [27.20%] patients were prescribed NNToC and reported a smaller MMSE reduction after two years compared to the 1,290 [72.80%] patients who were not (1.90 [0.71] vs 3.78 [0.80], respectively; P=0.05). (D) Joint plot of (A) (B) and (C) showing comparison together.

Table S10. Quantified Mini Mental State Examination (MMSE) score changes when ridge regression, random forest and one-dimensional Convolutional Neural Network (1D CNN) were used for drug recommendation. External validation on Oxford Health NHS Foundation Trust (OHFT). Unit: point. SD: standard deviation. NNToC: neural network treatment of choice.

|  | Cohort | 0.25 years | 0.5 years | 1 year | 1.5 years | 2 years |
| --- | --- | --- | --- | --- | --- | --- |
| Ridge regression | Patients prescribed NNToC, N=101, mean [SD] | 0.05 [0.28] | -0.26 [0.51] | -0.88 [0.69] | -1.35 [0.68] | -1.83 [0.69] |
|  | Patients prescribed non-NNToC, N=1,671, mean [SD] | 0.05 [0.27] | -0.11 [0.53] | -1.21 [0.68] | -2.64 [0.70] | -4.07 [0.69] |
| Random forest | Patients prescribed NNToC, N=708, mean [SD] | 0.17 [0.12] | -0.02 [0.26] | -0.66 [0.64] | -1.24 [0.72] | -1.76 [0.49] |
|  | Patients prescribed non-NNToC, N=1,064, mean [SD] | -0.11 [0.12] | -0.29 [0.27] | -1.25 [0.51] | -2.57 [0.69] | -3.92 [0.70] |
| One-dimensional Convolutional Neural Network (1D CNN) | Patients prescribed NNToC, N=482, mean [SD] | 0.15 [0.27] | -0.11 [0.61] | -0.79 [0.68] | -1.36 [0.71] | -1.90 [0.71] |
|  | Patients prescribed non-NNToC, N=1,290, mean [SD] | -0.06 [0.28] | -0.22 [0.62] | -1.12 [0.77] | -2.43 [0.80] | -3.78 [0.80] |

# Oversampling Patients Prescribed Minority Drugs for Model Training (Table S11, Figure S7, Table S12)

Table S11. Implementation details of the Long Short Term Memory (LSTM) model for the oversampled data.

| Structure | Fully connected layer (FC1) + LSTM layer (LSTM1) + LSTM layer (LSTM2) + fully connected layer (FC2) |
| --- | --- |
| Hyperparameter | FC1 units: 128  FC1 activation: tanh  FC1 dropout: 0.5  LSTM1 units: 50  LSTM1 activation: tanh  LSTM1 dropout: 0.5  LSTM2 units: 10  LSTM2 activation: tanh  LSTM2 dropout: 0.5  FC2 units: 1  FC2 activation: sigmoid  Learning rate: 0.0002  Optimiser: Adam  Loss: mean squared error (MSE)  Early stopping monitor: mean absolute error (MAE)  Early stopping patience: 200 |


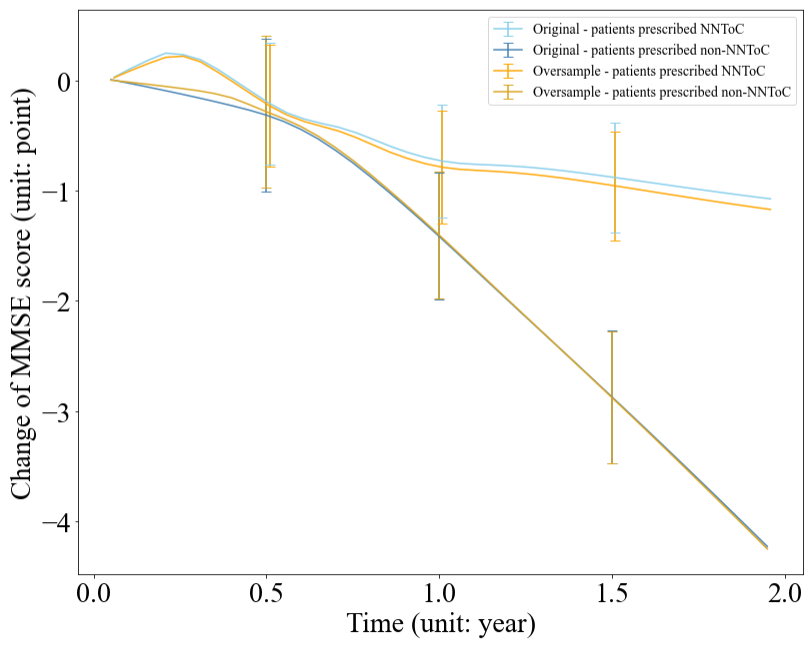


Figure S7. Change of Mini Mental State Examination (MMSE) score over time when the Long Short Term Memory (LSTM) model was trained on the oversampled data. Patients were grouped by whether they were prescribed neural network treatment of choice (NNToC) according to the LSTM model. The X-axis represents the duration of taking a particular medication, where $x=0$ means the time from when a medication was prescribed on a randomly selected visit. Data shown are mean values, with error bars indicating standard deviation. External validation on Oxford Health NHS Foundation Trust (OHFT). The model performed very similarly to the one trained on the original dataset. 243 [13.71%] patients were prescribed NNToC and reported a smaller MMSE reduction after two years compared to the 1,529 [86.29%] patients who were not (1.17 [0.50] vs 4.25 [0.60], respectively; P=0.01).

Table S12. Quantified Mini Mental State Examination (MMSE) score changes when the Long Short Term Memory (LSTM) model was trained on the oversampled data. External validation on Oxford Health NHS Foundation Trust (OHFT). Unit: point. SD: standard deviation. NNToC: neural network treatment of choice.

| Cohort\Year | 0.25 | 0.5 | 1 | 1.5 | 2 |
| --- | --- | --- | --- | --- | --- |
| Patients prescribed NNToC, N=243, mean [SD] | 0.21 [0.22] | -0.13 [0.50] | -0.75 [0.52] | -0.93 [0.50] | -1.17 [0.50] |
| Patients prescribed non-NNToC, N=1,529, mean [SD] | -0.05 [0.28] | -0.22 [0.62] | -1.26 [0.59] | -2.73 [0.60] | -4.25 [0.60] |

# Training a Single Model to Predict Both Scores Using Multitask Learning (Table S13, Figure S8, Table S14)

Table S13. Implementation details of the Long Short Term Memory (LSTM) model for multitask learning. FC: fully connected.

| Structure | FC layer (FC1) + LSTM layer (LSTM1) + LSTM layer (LSTM2) + $\left\{ \begin{aligned} FC layer \left( FC2 \right)+FC layer (FC4) \\ FC layer \left( FC3 \right)+FC layer (FC5) \end{aligned} \right.$ |
| --- | --- |
| Hyperparameter | FC1 units: 128  FC1 activation: tanh  LSTM1 units: 50  LSTM1 activation: tanh  LSTM2 units: 50  LSTM2 activation: tanh  LSTM2 dropout: 0.4  FC2/FC3 units: 32  FC2/FC3 activation: tanh  FC2/FC3 dropout: 0.5  FC4/FC5 units: 1  FC4/FC5 activation: sigmoid  Learning rate: 0.0002  Optimiser: Adam  Loss: mean squared error (MSE) of non-missing labels  Early stopping monitor: mean absolute error (MAE)  Early stopping patience: 200 |


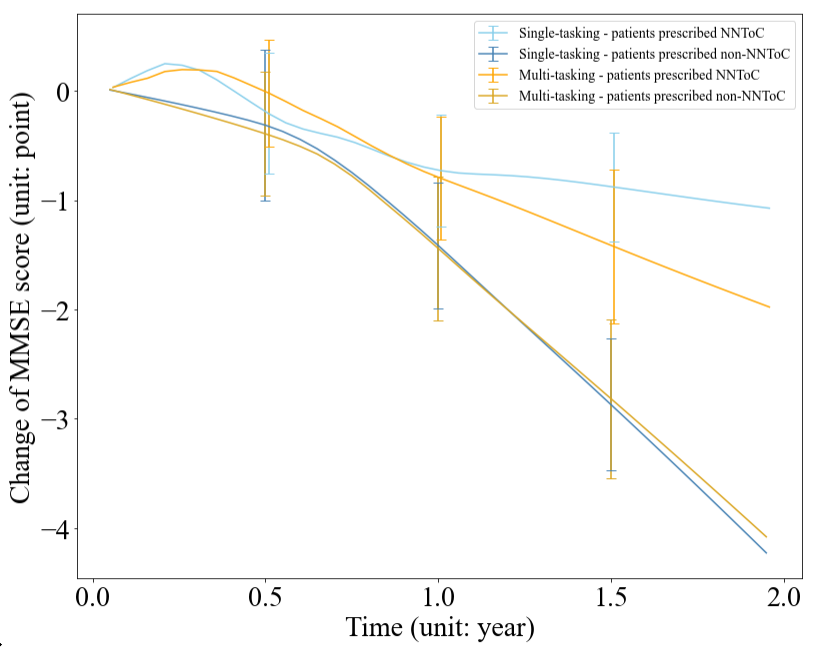

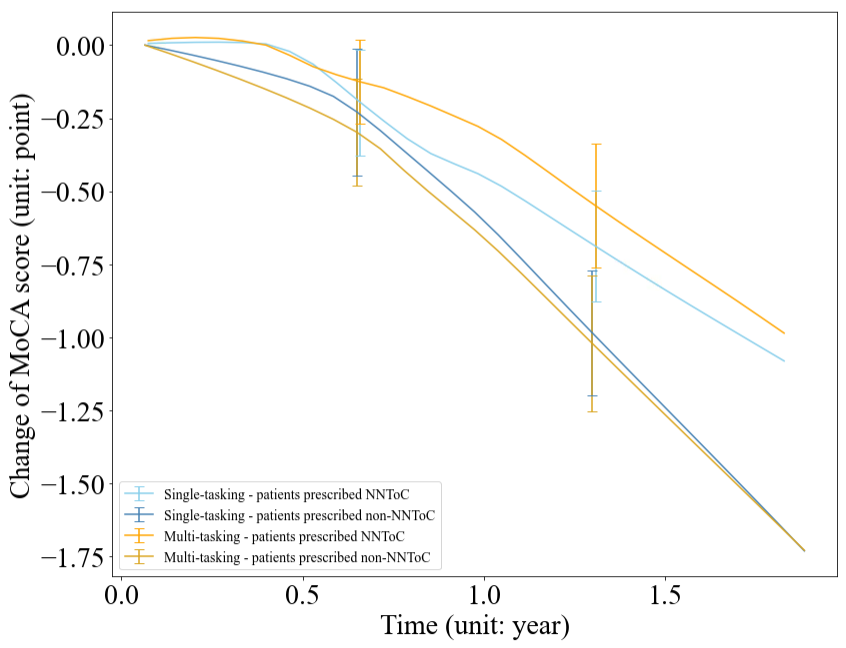


A

B

Figure S8. Change of Mini Mental State Examination (MMSE) and Montreal Cognitive Assessment (MoCA) score over time when recommendations were given by a single Long Short Term Memory (LSTM) model developed using multitask learning. Patients were grouped by whether they were prescribed neural network treatment of choice (NNToC) according to the model. The X-axis represents the duration of taking a particular medication, where $x=0$ means the treatment initiation time. Data shown are mean values, with error bars indicating standard deviation. External validation on Oxford Health NHS Foundation Trust (OHFT). (A) Change of MMSE score. 186 [10.50%] patients were prescribed NNToC and reported a smaller MMSE reduction after two years compared to the 1,586 [89.50%] patients who were not (1.98 [0.70] vs 4.08 [0.73], respectively; P=0.04). (B) Change of MoCA score. 202 [17.92%] patients were prescribed NNToC and reported a smaller MMSE reduction after two years compared to the 925 [82.08%] patients who were not (0.93 [0.21] vs 1.57 [0.23], respectively; P=0.08).

Table S14. Quantified Mini Mental State Examination (MMSE) and Montreal Cognitive Assessment (MoCA) score changes when recommendations were given by a single Long Short Term Memory (LSTM) model developed using multitask learning. Unit: point. SD: standard deviation. NNToC: neural network treatment of choice.

|  | Cohort | 0.25 years | 0.5 years | 1 year | 1.5 years | 2 years |
| --- | --- | --- | --- | --- | --- | --- |
| Change of MMSE | Patients prescribed NNToC, N=186, mean [SD] | 0.18 [0.20] | 0.05 [0.44] | -0.74 [0.55] | -1.36 [0.69] | -1.98 [0.70] |
|  | Patients prescribed non-NNToC, N=1,586, mean [SD] | -0.12 [0.23] | -0.34 [0.51] | -1.30 [0.65] | -2.68 [0.72] | -4.08 [0.73] |
| Change of MoCA | Patients prescribed NNToC, N=202, mean [SD] | 0.02 [0.06] | -0.07 [0.11] | -0.32 [0.18] | -0.89 [0.19] | NA |
|  | Patients prescribed non-NNToC, N=925, mean [SD] | -0.08 [0.07] | -0.22 [0.15] | -0.71 [0.21] | -1.32 [0.21] | NA |

# Evaluating the Impact of Training the Model with Fewer Observations per Patient (Table S15, Figure S9, Table S16)

Table S15. Implementation details of the Long Short Term Memory (LSTM) model when the model was trained with fewer observations per patient.

| Structure | Fully connected layer (FC1) + LSTM layer (LSTM1) + LSTM layer (LSTM2) + fully connected layer (FC2) |
| --- | --- |
| Hyper parameters for both 3 observations and 2 observations | FC1 units: 128  FC1 activation: tanh  LSTM1 units: 50  LSTM1 activation: tanh  LSTM2 units: 10  LSTM2 activation: tanh  LSTM2 dropout: 0.5  FC2 units: 1  FC2 activation: sigmoid  Learning rate: 0.0002  Optimiser: Adam  Loss: mean squared error (MSE)  Early stopping monitor: mean absolute error (MAE)  Early stopping patience: 200 |


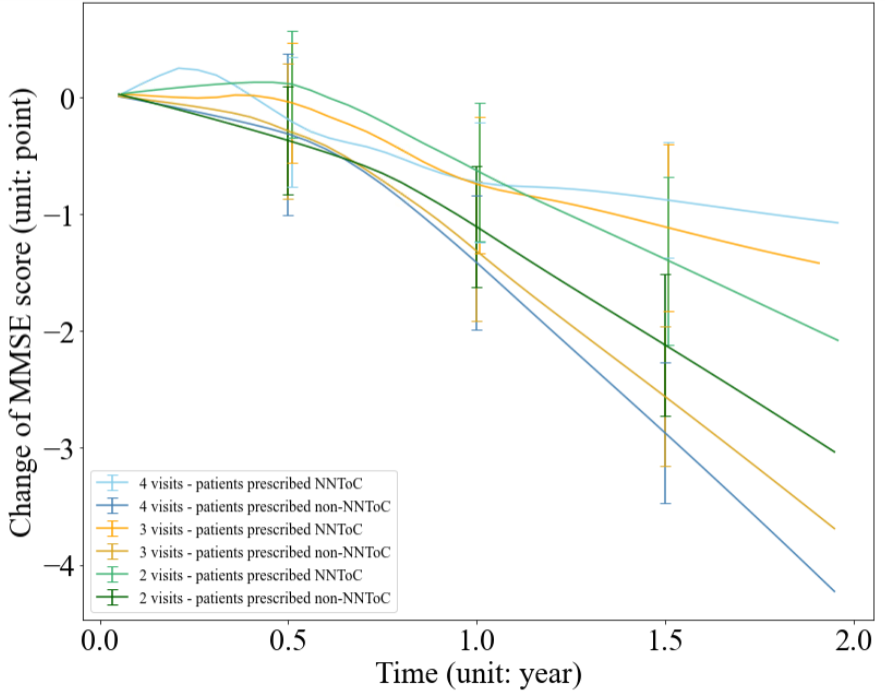


Figure S9. Change of Mini Mental State Examination (MMSE) score over time when the Long Short Term Memory (LSTM) model was trained with fewer observations per patient. Patients were grouped by whether they were prescribed neural network treatment of choice (NNToC) according to the model. The X-axis represents the duration of taking a particular medication, where $x=0$ means the treatment initiation time. Data shown are mean values, with error bars indicating standard deviation. External validation on Oxford Health NHS Foundation Trust (OHFT). If the model was trained using 3 observations. 170 [9.59%] patients were prescribed NNToC and reported a smaller MMSE reduction after two years compared to the 1,602 [90.41%] patients who were not (1.53 [0.71] vs 3.69 [0.60], respectively; P=0.04). If the model was trained using 2 observations, 786 [44.36%] patients were prescribed NNToC and reported a smaller MMSE reduction after two years compared to the 986 [55.64%] patients who were not (2.08 [0.65] vs 3.03 [0.61], respectively; P=0.06).

Table S16. Quantified Mini Mental State Examination (MMSE) score changes when the Long Short Term Memory (LSTM) model was trained with fewer observations per patient. Unit: point. SD: standard deviation. NNToC: neural network treatment of choice.

|  | Cohort | 0.25 years | 0.5 years | 1 year | 1.5 years | 2 years |
| --- | --- | --- | --- | --- | --- | --- |
| 3 observations | Patients prescribed NNToC, N=170, mean [SD] | -0.01 [0.21] | -0.01 [0.46] | -0.70 [0.57] | -1.07 [0.70] | -1.53 [0.71] |
|  | Patients prescribed non-NNToC, N=1,602, mean [SD] | -0.05 [0.23] | -0.22 [0.52] | -1.18 [0.60] | -2.44 [0.60] | -3.69 [0.60] |
| 2 observations | Patients prescribed NNToC, N=786, mean [SD] | 0.08 [0.18] | 0.13 [0.41] | -0.56 [0.58] | -1.32 [0.71] | -2.08 [0.65] |
|  | Patients prescribed non-NNToC, N=986, mean [SD] | -0.10 [0.18] | -0.32 [0.41] | -1.01 [0.51] | -2.02 [0.60] | -3.03 [0.61] |

# Permutation Feature Importance (Figure S10, Table S17)

Permutation feature importance measures the decrease of a model’s performance when a single predictor in tabular formatted data is randomly shuffled. Specifically, the model was fitted on the training data as usual to learn a latent pattern between predictors and outcomes. We then fitted the trained model on the testing data (OHFT external validation set) and generated predictions. Next, we randomly shuffled the values of a single predictor in the testing data 1,000 times, where we broke the mapping between predictors and outcomes. Since the mapping was broken, the model yielded worse predictions. This was seen as a decrease in the model’s performance. Since our model directly outputted cognitive scores, we used the increase of mean absolute error (MAE) to measure the performance decrease. A more substantial change indicates a more predictive feature.


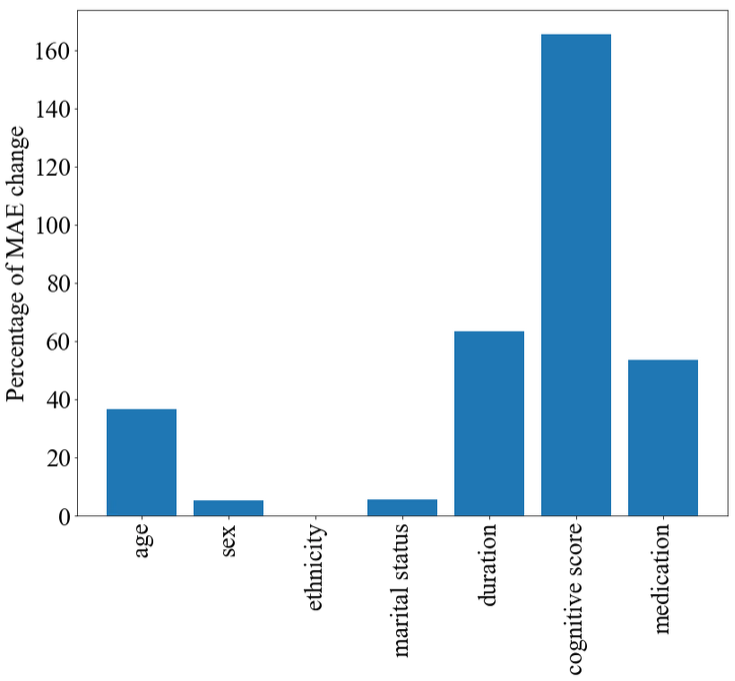

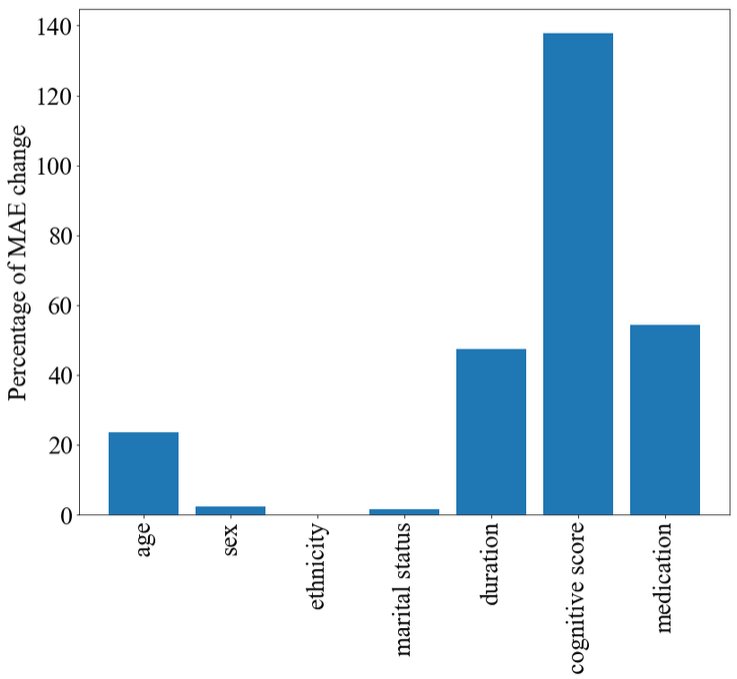


1. Mini Mental State Examination (MMSE)
2. Montreal Cognitive Assessment (MoCA)

Figure S10. Permutation feature importance of the Long Short Term Memory (LSTM) model. The X-axis represents predictors and the Y-axis represents the percentage increase in mean absolute error (MAE). Each predictor was randomly shuffled 1,000 times (A) Mini Mental State Examination (MMSE) model. (B) Montreal Cognitive Assessment (MoCA) model.

Table S17. Permutation feature importance scales of the Long Short Term Memory (LSTM) model. MAE: mean absolute error.

| **Predictor** | **Mean percentage increase in MAE** | |
| --- | --- | --- |
|  | Mini Mental State Examination (MMSE) | Montreal Cognitive Assessment (MoCA) |
| Age | 36.65 | 23.76 |
| Sex | 5.31 | 2.30 |
| Ethnicity | 0.10 | 0.08 |
| Marital status | 5.71 | 1.62 |
| Duration | 63.46 | 47.41 |
| Cognitive score | 165.52 | 137.80 |
| Medication | 53.76 | 54.25 |
